# Porcine Circovirus Type 3 Cap Inhibits Type I Interferon Induction Through Interaction With G3BP1

**DOI:** 10.3389/fvets.2020.594438

**Published:** 2020-12-17

**Authors:** Pengfei Zhang, Hanqin Shen, Xianhui Liu, Shuangyun Wang, Yanling Liu, Zheng Xu, Changxu Song

**Affiliations:** ^1^College of Animal Science and National Engineering Center for Swine Breeding Industry, South China Agriculture University, Guangzhou, China; ^2^Wen's Foodstuff Group Co. Ltd, Guangdong Enterprise Key Laboratory for Animal Health and Environmental Control, Yunfu, China

**Keywords:** porcine circovirus, PCV3, IFN signaling, capsid protein, cGAS, G3BP1

## Abstract

Porcine circovirus 3 (PCV3) infections cause clinical diseases similar to those seen in porcine circovirus 2 (PCV2) infections. It is unclear whether PCV3 infections can also cause immunosuppression like that seen with PCV2. Here, we report that Cap inhibits DNA-induced IFN-β mRNA transcription and IFN promoter activation. Cap was also found to inhibit cyclic GMP-AMP (cGAMP) synthase (cGAS) binding to interferon-stimulating DNA (ISD). Immunoprecipitation and mass spectrometry were used to identify cellular interaction partners of Cap. Cap interacted with G3BP1 and inhibited the interaction between GTPase-activating protein-(SH3 domain)-binding protein 1 (G3BP1) and cGAS. Furthermore, the destruction of endogenously expressed G3BP1 by siRNA significantly reduced IFN promoter activation, and phosphorylation of tank-binding kinase 1 (TBK1) was induced by ISD. Overexpression of G3BP1 attenuated the inhibition of ISD binding of cGAS by Cap and promoted phosphorylation of TBK1 and IRF3 induced by ISD. Collectively, our results show that the interaction between Cap and G3BP1 prevents cGAS from recognizing DNA, thereby inhibiting the IFN production.

## Introduction

Porcine circovirus 3 (PCV3), a DNA virus, has a genome of only 2 kb in size with two main open reading frames (ORFs) encoding the replicase protein and capsid (Cap) protein (1). Currently, four types of porcine circoviruses (PCVs) are known (2). While porcine circovirus 1 (PCV1) is considered nonpathogenic to pigs (3), porcine circovirus 2 (PCV2) is the pathogen responsible for the multiple clinical manifestations of porcine circovirus-associated diseases, such as nephropathy syndrome (PDNS) and postweaning multisystemic wasting syndrome and porcine dermatitis (4–6), which threaten the global swine industry. In 2015, a third disease, PCV3, was first identified in the USA from a case of PDNS (7). In recent years, PCV3 has been reported in multiple countries, where it is often associated with PDNS, multisystemic inflammation, congenital tremors, and respiratory disease (8–12). In 2019, porcine circovirus 4 (PCV4) was detected in diseased pigs with PDNS, respiratory, and enteric signs (13). PCV3 was detected in a coinfection with PCV2 and porcine epidemic diarrhea virus from a severe case of diarrheal disease in China, and the authors reported a triple infection rate of 48.68% (14). PCV3 was first isolated in 2020 in China in perinatal and reproductive sows (15). Therefore, recent reports suggest that more attention should be given to PCV3-associated disease.

The innate immune response is the first line of antiviral defense to limit viral spread. DNA sensors, one type of pattern recognition receptors, recognize pathogen-associated molecular patterns (PAMPs) and trigger downstream signaling to elicit innate immune responses and to further activate the adaptive immune system (16, 17). Cyclic GMP-AMP (cGAMP) synthase (cGAS) is the host's cytosolic DNA sensor. The foreign DNA molecules introduced into the cytoplasm after infection with a DNA virus are PAMPs. When PAMPs are recognized by cGAS, the cGAMP second messenger binds to the adaptor protein STING (stimulator of IFN genes), which directs tank-binding kinase 1 (TBK1)-mediated interferon regulatory factor 3 (IRF3) activation and type I interferon (IFN) production (18, 19). GTPase-activating protein-(SH3 domain)-binding protein 1 (G3BP1), an antiviral protein, promotes multiple innate antiviral immune responses (20). Many viruses have been shown to target G3BP1 and inhibit stress granule formation (20, 21). Recently, G3BP1 was shown to promote DNA binding and activate cGAS (22). In the present study, we report that the Cap protein is able to inhibit DNA-induced IFN-β production, and that Cap interacts with G3BP1 to prevent cGAS from recognizing DNA. Our findings will enhance current understanding about the mechanism underlying the immune escape response used by PCV.

## Materials and Methods

### Cells and Reagents

Human embryonic kidney (HEK) 293T cells and porcine kidney (PK) 15 cells were maintained in Dulbecco's modified Eagle's medium (DMEM) supplemented with 10% fetal bovine serum with antibiotics (100 units/ml penicillin, and 100 μg/ml streptomycin).

### Plasmids

The Cap expression plasmid was constructed according to a previously described method (23). ORF2 (GenBank accession number: KY418606), which was acquired by standard PCR with primers (Table 1), was subcloned into the pcDNA3.1-3×FLAG vector using standard molecular biological techniques. DNA fragments from the full-length and mutated G3BP1 were acquired by standard PCR with primers (Table 1) and subcloned into the pcDNA3.1-HA vector. The cGAS gene was acquired by standard PCR with primers (Table 1), and then subcloned into vector pcDNA3.1-3×FLAG.

**Table 1:** The primer used.

**Name**	**Sequences (5' to 3')**	**Use**
Cap-flag-F	CGCGGATCCATGAGACACAGACCTATATTCA (BAMH I)	Vector construction
Cap-flag-R	CCGGAATTCTTAGAGAACTGACTTGTAACGA (ECOR I)	
Cap-HA-F	CCGGAATTCGGATGAGACACAGACCTATATTCA	Vector construction
Cap-HA-R	CGGGGTACCTTAGAGAACTGACTTGTAACGA	
pG3BP1-R	CTAGGCTTCTCCATAACCATCCGAATTCGGGCCTCCATGG	Vector construction
pG3BP1-F	CTTACTCCAAGGCAGTGAGGTACCGCGGCCGCGGGGAT	
G3BP1-R	TCACTGCCTTGGAGTAAGGC	Vector construction
G3BP1-F	ATGGTTATGGAGAAGCCTAGT	
cGAS-F	ACGATGACAAGCTTGGTACCATGCAGCCTTGGCACGGAAA	Vector construction
cGAS-R	GGACTAGTGGATCCGAGCTCTCAAAATTCATCAAAAACTGGAA	
STING-F	ACGATGACAAGCTTGGTACCATGCCCCACTCCAGCCTGCA	Vector construction
STING-R	GGACTAGTGGATCCGAGCTCTCAAGAGAAATCCGTGCGGAGA	
pFLAG-F	GAGCTCGGATCCACTAGTCCA	Vector construction
pFLAG-R	GGTACCAAGCTTGTCATCGTCA	
G3BP1-1-F	CCCGAATTCGGGGCTTTGTCACGGAGCCTCAG	Vector construction
G3BP1-1-R	ACAAAGCCCCGAATTCGGGCCTCCATGGCCA	
G3BP1-2-F	GGTCTTTGGTGCTCCTGAGGATGTTCAGAAGAG	Vector construction
G3BP1-2-R	CCTCAGGAGCACCAAAGACCTCATCTTGGTATCTGAA	
G3BP1-3-F	GGAAGAAGCTCTCTTCATTGGCAACCTGCCCCATGAGG	Vector construction
G3BP1-3-R	CAATGAAGAGAGCTTCTTCCAATACTGGCTCAGAC	
G3BP1-4-F	CCCTGACAGCCACCAGGTGGAAGAAAAGAAGACCCGAG	Vector construction
G3BP1-4-R	TCCACCTGGTGGCTGTCAGGGTGTCTCACGATTCTTCGA	
G3BP1-5-F	GGTCCGTCTGAATTGAGGTACCGCGGCCGCGGGGATCCAGA	Vector construction
G3BP1-5-R	GTACCTCAATTCAGACGGACCTCACCTCTGAACATGATGG	
p-GAPDH-F	ACATGGCCTCCAAGGAGTAAGA	Real-time PCR
p-GAPDH-R	GATCGAGTTGGGGCTGTGACT	
pIFN-β-F	CATCCTCCAAATCGCTCTCC	Real-time PCR
pIFN-β-R	CTGACATGCCAAATTGCTGC	
NC-Sense	UUCUCCGAACGUGUCACGUTT	siRNA ologo sequences targeting to G3BP1
NC-Antisense	ACG UGA CAC GUU CGG AGA ATT	
1#-Sense	GGGAAUUUGUGAGACAGUATT	
1#-Antisence	UACUGUCUCACAAAUUCCCTT	
2#-Sence	GCCAGUAUUGGAAGAAGCUTT	
2#-Antisense	AGCUUCUUCCAAUACUGGCTT	
3#-Sense	GAUACCACCUCAUGUUGUUTT	
3#-Antisense	AACAACAUGAGGUGGUAUCTT	

### Quantitative RT-PCR

PK15 cells seeded on coverslips in 24-well plates were transfected with the pcDNA3.1-3×FLAG empty vector or with FLAG-Cap. At 24 h post-transfection (hpt), the cells were stimulated with 2 μg/ml ISD for another 6 h. The cells were harvested, and total cellular RNA was extracted using the MiniBEST Universal RNA Extraction Kit (Takara, Japan) according to the manufacturer's instructions. cDNA was generated by reverse transcription using Prime Script™ RT Master Mix (Takara). QRT-PCR was performed using the Maxima SYBR Green qPCR Master Mix Kit (Thermo Scientific) in the Applied Biosystems StepOnePlus Real-Time PCR System (Life Technologies). The gene expression levels were normalized to those of glyceraldehyde 3-phosphate dehydrogenase. The amplification primers are listed in Table 1.

### Dual-Luciferase Reporter Assay

PK15 cells seeded on coverslips in 24-well plates were co-transfected with 100 ng of IFN-Luc, 100 ng of TK-Luc and FLAG-Cap, or the pcDNA3.1-3×FLAG empty vector. At 24 hpt, the cells were stimulated with 2 μg/ml ISD or 1 μg Poly (dA:dT) for another 12 h, and the cell lysates were prepared from them for luciferase analysis using the luciferase enzyme assay system (Promega, China) according to the manufacturer's instructions and as previously described (23).

### Immunoprecipitation and Immunoblotting

After transfection or stimulation, the cell lysates were incubated overnight with appropriate antibodies at 4°C in a rotator. Protein samples mixed with antibodies were incubated with beads for 1 h at 4°C in a rotator, and the beads were washed three times with TBST buffer and eluted with 2× sodium dodecyl sulfate (SDS)-loading buffer. The cell lysates or precipitates were loaded onto 10 or 12% polyacrylamide gels, transferred to nitrocellulose membranes (Millipore, Shanghai, China), and further incubated with primary antibodies. The antibodies used in this study are listed as follows and were purchased from Cell Signaling Technology (Danvers, MA): HRP-linked anti-mouse IgG (7076) antibodies, HA-Tag (HRP conjugate) (14031), anti-HA-Tag (C29F4), HRP-linked anti-rabbit IgG (7074), anti-TBK1, anti-pTBK1, anti-IRF3, and anti-pIRF3. Anti-FLAG M2 (F3165) antibody and anti-β-actin (A1978) antibody were purchased from Sigma. The G3BP1 mouse monoclonal antibody was purchased from Proteintech, and the cGAS/C6orf150 (4E5) mouse monoclonal antibody was obtained from Zen-Bioscience (Chengdu, China).

### Biotinylated-DNA IP

HEK 293T cells were co-transfected with FLAG-cGAS and FLAG-Cap or the empty expression vector using Lipofectamine LTX Reagent at 80% cell confluence and following the manufacturer's instructions. After 24 h, the transfected cells were transfected with 2 μg biotin-labeled DNA (ISD) (5′-biotin-TACAGATCTACTAGTGATCTATGACTGATCTGTACATGATCTACA-3′) for another 2 h, and the cells were washed three times with phosphate-buffered saline and lysed with RIPA buffer (20 mM Tris-HCl pH 7.4, 1 mM EDTA, 150 mM NaCl, and 1% NP40) containing 5% glycerol and protease inhibitors (Complete Mini, Roche). The lysates were incubated with 20 μl Pierce™ High Capacity Streptavidin Agarose (Thermo Scientific) for 1 h at 4°C. The beads were recovered by centrifugation (3,000 × *g*, 1 min, 4°C), and the supernatant was discarded. Immunoprecipitation (IP) preparations were washed three times with lysis buffer, and the beads were recovered by centrifugation (3,000 × *g*, 1 min, 4°C). Proteins were eluted by boiling in 2 × SDS polyacrylamide gel electrophoresis (PAGE) loading buffer. The precipitated samples were analyzed by SDS-PAGE followed by immunoblotting.

### Statistical Analysis

All the results in the figures are expressed as the means ± standard errors of the means (SEMs), and all analyses used GraphPad Prism 8.1 (GraphPad Software, Inc.). Differences were considered significant when the *p* value was <0.05, and the *p* values are as follows: ^*^p < 0.05, ^**^p < 0.01, and ^***^p < 0.005. The dates from at least three independent experiments were combined and analyzed by two-way ANOVA or *t* tests.

## Results

### PCV3 Cap Inhibits DNA-Induced IFN-β mRNA Transcription and IFN Promoter Activation

**Figure 1:**
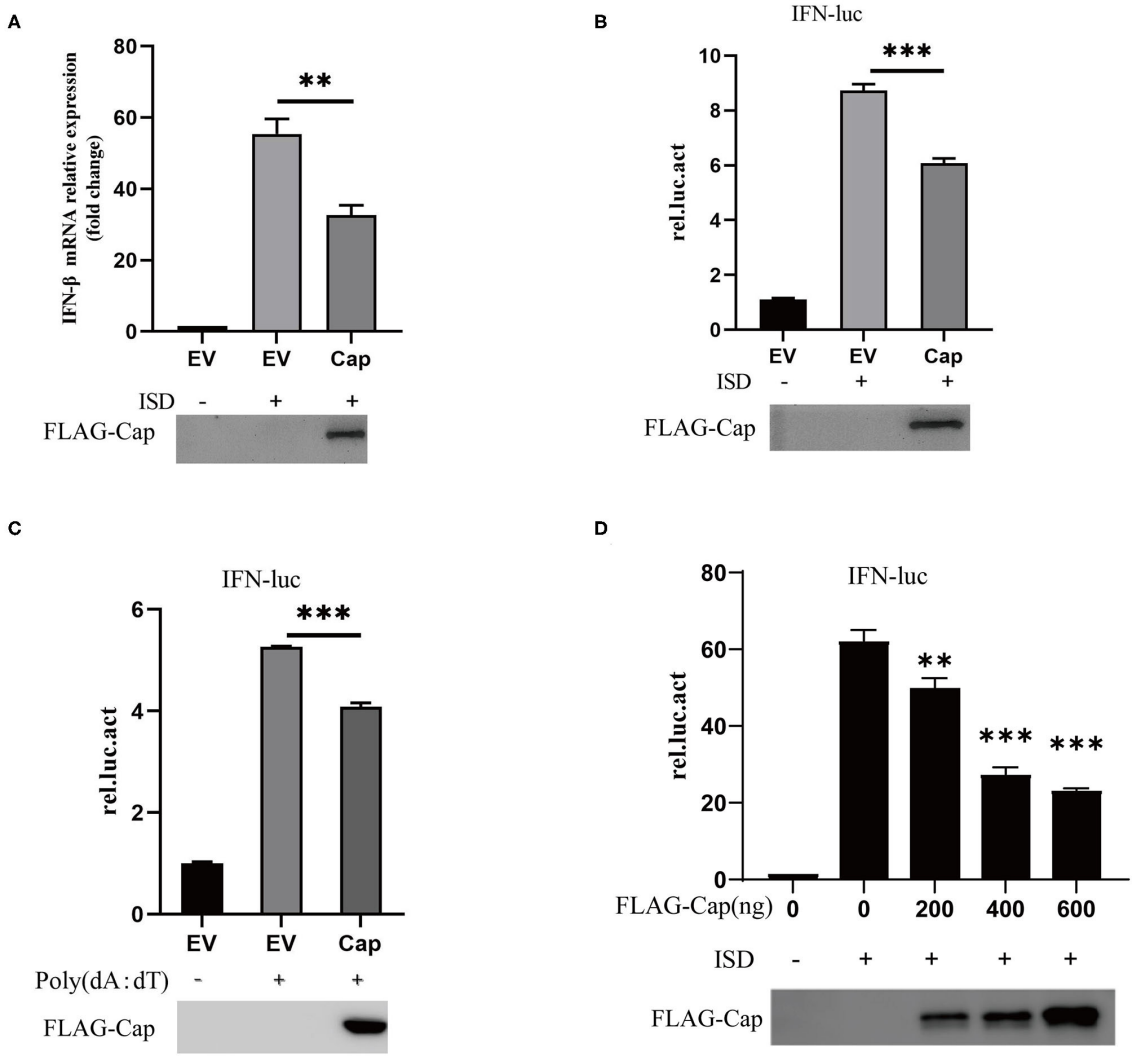
PCV3 Cap inhibits DNA-induced IFN-β production. **(A)** qPCR analysis of IFN-β mRNA in PK15 cells transfected with FLAG empty vector (EV) or the FLAG-Cap expression vector and stimulated with 2 μg/ml of ISD for 6 h at 24 h post-transfection. **(B–D)** Cap inhibits DNA-induced IFN promoter activation. Cells were co-transfected with 100 ng IFN-luc, 50 ng TK-luc, and 500 ng FLAG-Cap or the EV. After 24 h, the cells were stimulated by transfection with 2 μg/ml ISD **(B)** or 1 μg Poly (dA:dT) **(C)** for another 12 h and then lysed with cell lysis buffer for a dual-luciferase assay. **(D)** PK15 cells were transfected with the indicated amount of the FLAG-Cap expression vector. The results represent data from three independent experiments, *p < 0.01; ***p < 0.001.

Type I IFNs play an important role in the host's innate immune response to viral infections and viruses can inhibit IFN production in different ways. To investigate whether Cap inhibits the production of type I IFN, PK15 cells were transfected with FLAG-Cap or an empty vector. At 24 hpt, cells were transfected with 2 μg/ml of ISD for another 6 h, total cellular RNA was extracted, and IFN-β mRNA levels were quantified by real-time PCR. As shown in Figure 1A, ISD was able to stimulate IFN-β mRNA transcription, whereas overexpression of Cap was found to significantly inhibit the ISD-induced transcription of IFN-β mRNA.

To investigate whether Cap inhibited ISD-induced type I IFN promoter activation, PK15 cells were co-transfected with 100 ng of IFN-Luc, 100 ng of TK-Luc and FLAG-Cap, or the empty vector. At 24 hpt, the cells were stimulated with 2 μg/ml ISD or 1 μg Poly (dA:dT) for another 12 h, and the cell lysates were prepared from them for luciferase or western blot analyses. The results showed that ISD and Poly (dA:dT) significantly enhanced the activity of the IFN promoter, but overexpression of Cap significantly suppressed the IFN promoter activity induced by DNA [ISD and Poly (dA:dT)], and the effect was dose dependent (Figures 1B–D). This indicates that PCV3 Cap can inhibit type I IFN signaling.

### PCV3 Cap Inhibits cGAS-Binding to DNA

**Figure 2:**
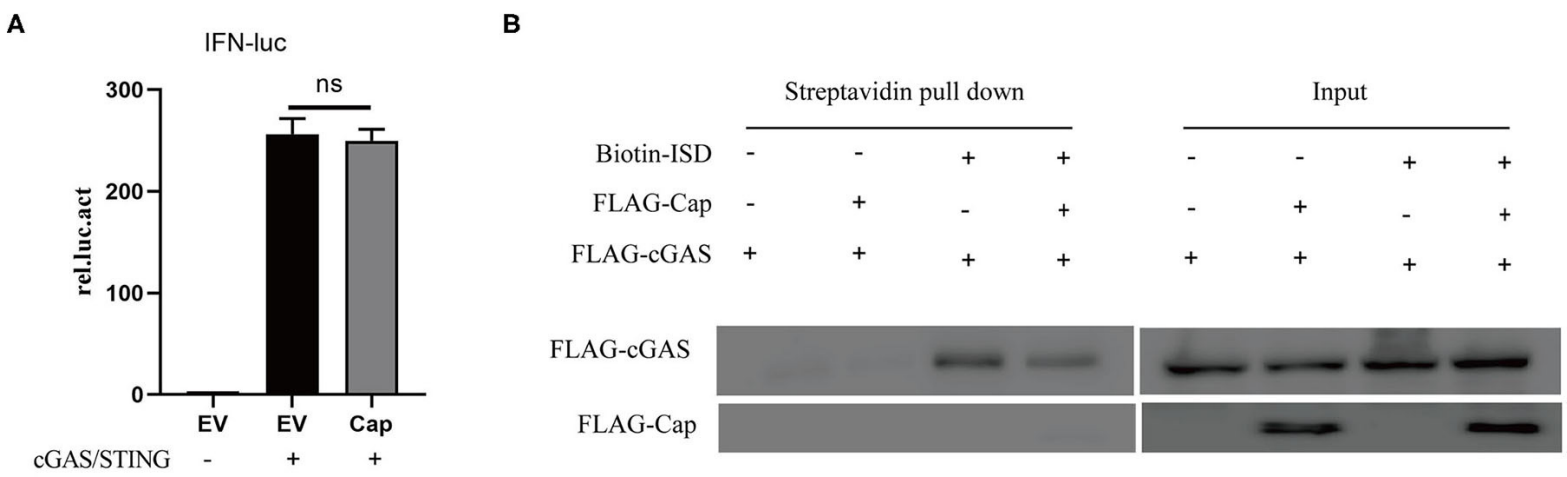
PCV3 Cap inhibits cGAS binding to DNA. **(A)** PCV3 Cap did not affect IFN promoter induction by cGAS and STING. HEK 293T cells were co-transfected with IFN-Luc, TK-Luc, FLAG-Cap, empty vector (EV), FLAG-cGAS, and FLAG-STING. At 24 hpt, the cell lysates were prepared for luciferase analysis. **(B)** Cap inhibits ISD binding of cGAS. HEK 293T cells were co-transfected with FLAG-cGAS, FLAG-Cap, or the empty vector (EV). At 24 hpt, the cells were transfected with biotin-ISD for another 2 h, and the cell lysates were prepared from them for biotin-ISD pull-down and immunoblotting analysis.

cGAS is an important cytosolic sensor for DNA, and the cGAS-STING pathway is very important for host defenses against viral infections (24). To further investigate whether Cap affects cGAS and STING-mediated IFN promoter activation, HEK 293T cells were co-transfected with IFN-Luc, TK-Luc, FLAG-Cap, empty vector, and FLAG-cGAS and FLAG-STING. The cell lysates were prepared for luciferase analysis at 24 hpt. The results showed that cGAS and STING-mediated IFN promoter activation were unaffected by Cap (Figure 2A). These results suggest that Cap inhibition of DNA-induced IFN production may affect the upstream region of cGAS-STING; therefore, we investigated whether Cap was able to inhibit cGAS binding to DNA. HEK 293T cells were co-transfected with FLAG-cGAS, FLAG-Cap, or empty vector. At 24 hpt, the cells were transfected with biotin-ISD for another 2 h, and the cell lysates were prepared for biotin-ISD pull-down and immunoblotting analysis. Cap was not co-precipitated with biotin-ISD, and the protein level of the cGAS co-precipitated with biotin-ISD was significantly lower in the transfected Cap group than in the empty vector group (Figure 2B), indicating that Cap might prevent DNA binding to cGAS and inhibit DNA-induced IFN production.

### Investigating the Interaction Between PCV3 Cap and G3BP1

**Figure 3:**
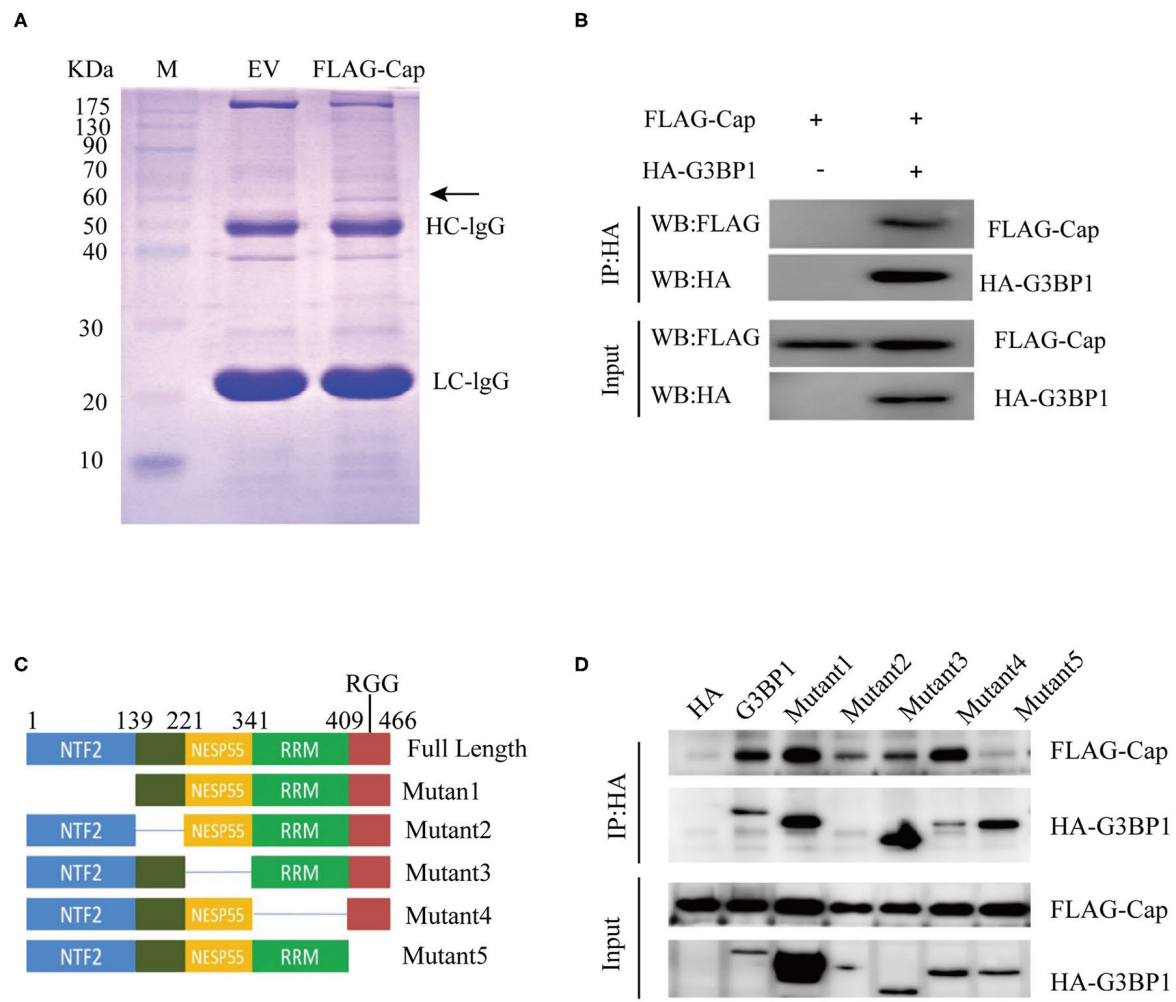
Identifying Cap-interacting proteins. **(A)** SDS-PAGE analysis of Cap pull-down samples. PK15 cells were transfected with FLAG-Cap or FLAG vector. The cells were lysed for immunoprecipitation (IP) with anti-FLAG antibody. The precipitates were analyzed by SDS-PAGE. **(B–D)** Interaction between Cap and G3BP1. HEK 293T cells were co-transfected with FLAG-Cap and HA-G3BP1 **(B)** or various HA-G3BP1 mutants **(D)**. The cells were lysed at 24 h post-transfection for IP using an anti-HA antibody, and the IP products were analyzed by western blotting. **(C)** Mapping the interacting regions between G3BP1 and Cap.

To further investigate the mechanism whereby PCV3 Cap inhibits IFN production, PK15 cells were transfected with FLAG-Cap or the empty vector for 24 h. The cells were then lysed and pulled down using an anti-FLAG antibody, and the eluent was collected and analyzed by SDS-PAGE. As shown in Figure 3A, compared with the empty vector group, the transfected Cap group produced a unique band in the 60-kDa range. This unique band was sent for mass spectrometry analysis. Most of the proteins pulled down by Cap are listed in Table 2. G3BP1, an important constituent molecule in stress granules, had the highest coverage value; therefore, we assessed whether Cap and G3BP1 could interact with each other.

**Table 2:** Identified proteins from immunoprecipitation reaction of Cap.

**Coverage**	**Description**
0.6402	G3BP stress granule assembly factor 1
0.5453	Eukaryotic translation initiation factor 2A
0.4395	Y-box binding protein 3
0.4202	Uncharacterized protein
0.3687	Insulin like growth factor 2 mRNA binding protein 2
0.3568	Heterogeneous nuclear ribonucleoprotein K
0.3549	G3BP stress granule assembly factor 2
0.3193	Prelamin-A/C
0.3138	T-complex protein 1 subunit gamma
0.3107	SWI/SNF related, matrix associated, actin dependent regulator of chromatin, subfamily d, member
0.263	Uncharacterized protein
0.2616	Uncharacterized protein
0.2482	Uncharacterized protein
0.2471	DEAD-box helicase 5
0.2402	Double stranded RNA-dependent protein kinase

HEK 293T cells were co-transfected with FLAG-Cap, HA-G3BP1, or the HA empty vector for 24 h, and then lysed and pulled down using anti-HA. As shown in Figure 3B, Cap interacted with G3BP1. G3BP1 contains four domains (25), so we sought to determine the domain(s) responsible for the G3BP1-Cap interaction by generating a series of deletions in G3BP1, and the interactions of these proteins with Cap were analyzed by a Co-IP assay. The interaction between G3BP1 and Cap was reduced after the RRG region was deleted (Figures 3C,D), indicating that RGG is necessary for the interaction of G3BP1 and Cap.

### G3BP1 Is Involved in DNA-Induced IFN Promoter Activation

**Figure 4:**
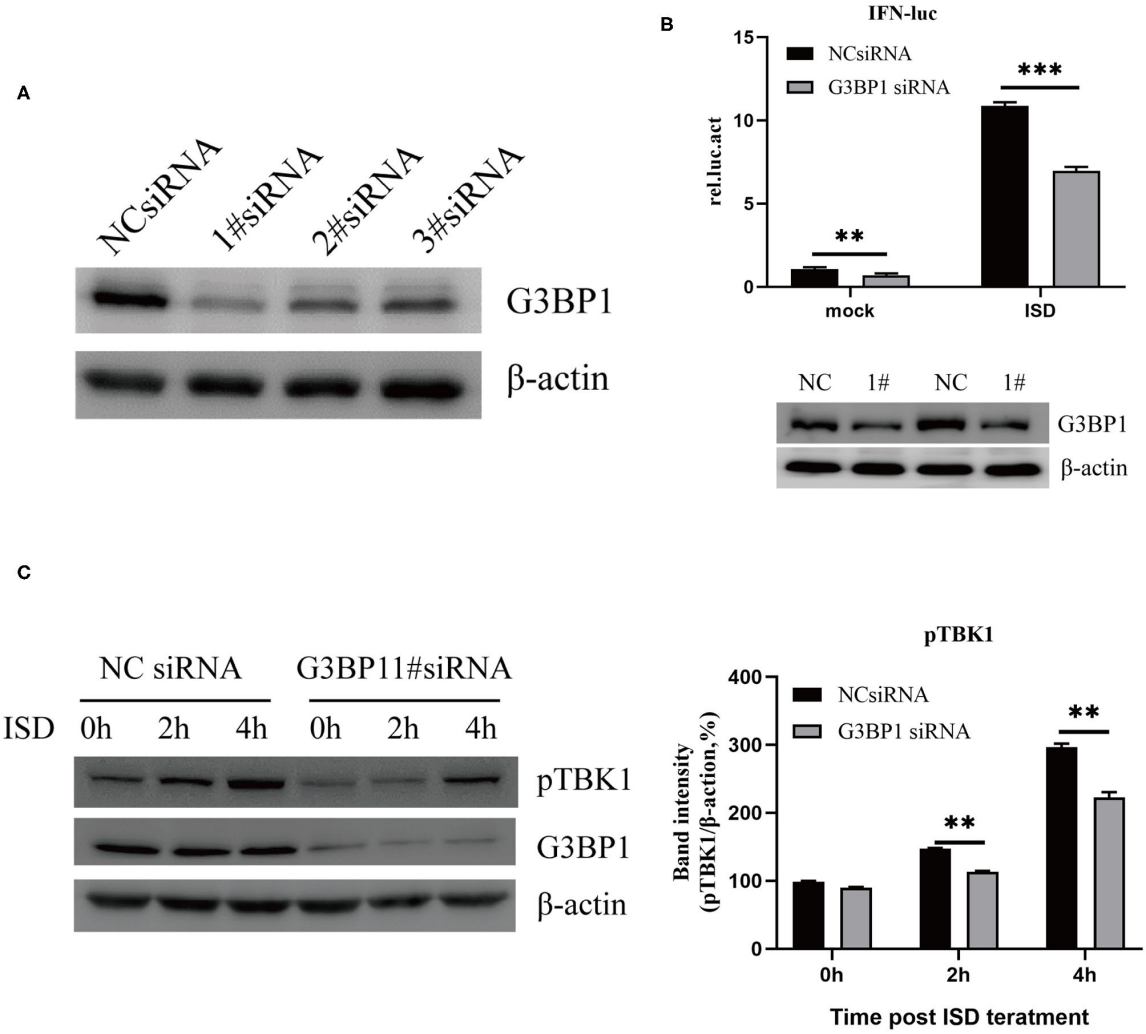
G3BP1 is involved in DNA-induced IFN promoter activation. **(A)** Western blotting analysis of G3BP1 expression after siRNA transfection. **(B)** Depletion of G3BP1 inhibits DNA-induced IFN promoter activation. PK15 cells were transfected with the negative control (NC) or G3BP1 #1 siRNA and the indicated reporter plasmids 24 h after siRNA transfection. The cells were then treated with ISD (2 μg/ml) for 12 h, and their luciferase activities were determined. G3BP1 and β-actin expression were detected by western blotting. **(C)** Depletion of G3BP1 inhibits DNA-induced TBK1 activation. PK15 cells transfected with NC or siRNA were treated with ISD, and 24 h after siRNA transfection, the cell lysates were analyzed by western blotting and the intensities of the pTBK1 and β-actin bands were quantified and normalized to β-actin. The results represent data from three independent experiments, **p < 0.01; ***p < 0.001.

Studies have reported that the cGAS-G3BP1 interaction promotes cGAS binding to DNA and supports cGAS-dependent IFN production (22). To study the effect of G3BP1 on ISD-induced IFN production, small interfering RNAs (siRNAs) targeting the G3BP1 gene were designed. PK15 cells were transfected with siRNAs for 24 h, and the cells were lysed and analyzed by western blotting, which showed that the siRNAs significantly reduced the expression of G3BP1 (Figure 4A). We then investigated the effect of silencing G3BP1 on ISD-induced IFN promoter activation. PK15 cells were transfected with NC siRNA or G3BP1 #1 siRNA, and at 24 hpt, the cells were co-transfected with IFN-Luc and TK-Luc, stimulated with 2 μg/ml ISD, and the cell lysates were prepared for luciferase analysis. The results showed that the proportion of G3BP1 in the siRNA-treated PK15 cells was significantly lower than in the NC-siRNA group, and that IFN promoter activation was significantly suppressed (Figure 4B). Western blots were used to analyze ISD-induced TBK1 phosphorylation in the PK15 cells transfected with G3BP1 #1 siRNA. Figure 4C shows that the PK15 cells transfected with G3BP1 #1 siRNA had significantly reduced ISD-induced TBK1 phosphorylation, indicating that G3BP1 is involved in ISD-induced IFN promoter activation.

### Cap Inhibits the Interaction Between cGAS and G3BP1

**Figure 5:**
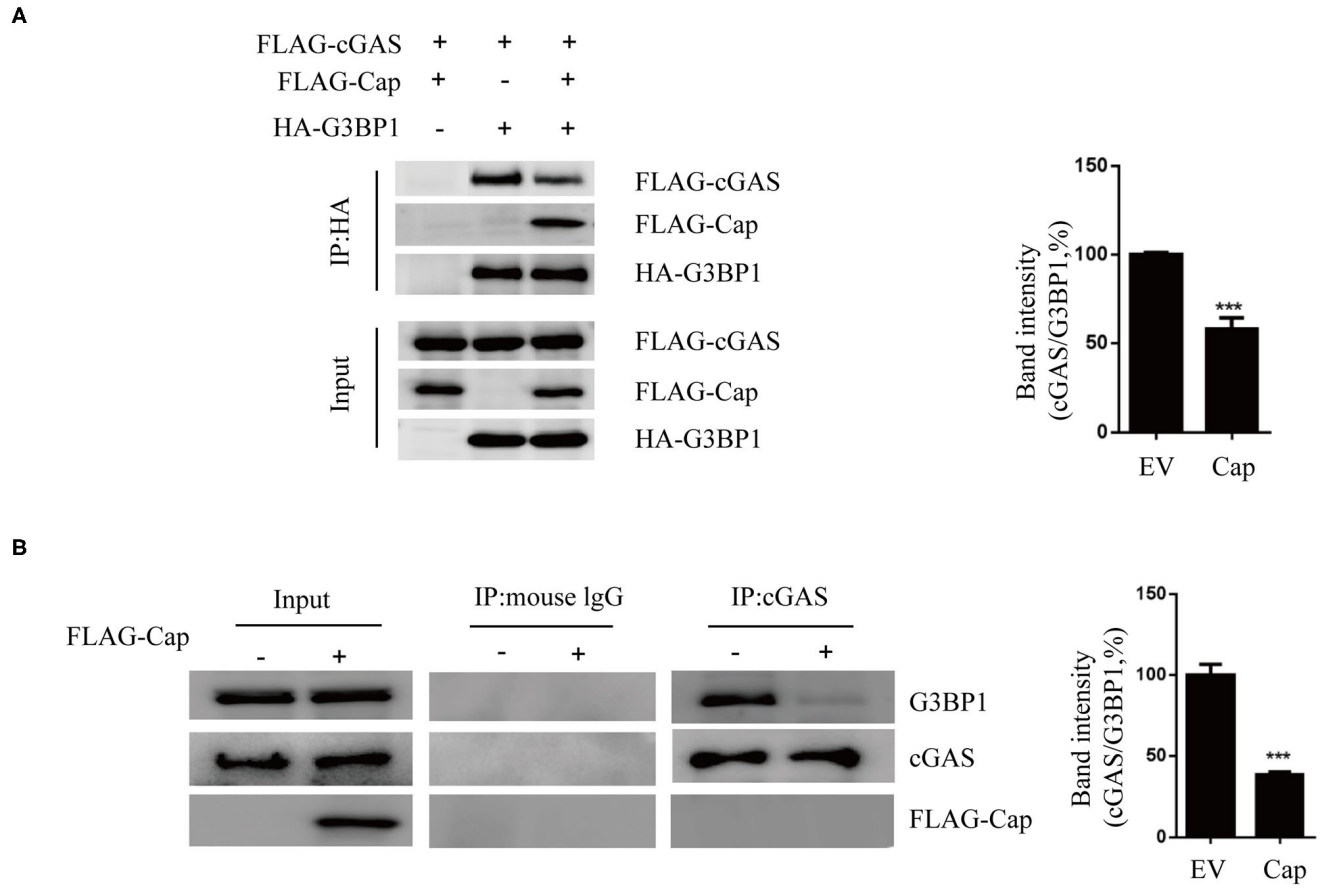
Cap inhibits the interaction between cGAS and G3BP1. **(A)** Cap reduced the amount of cGAS co-precipitated by G3BP1. HEK 293T cells were co-transfected with FLAG-cGAS, FLAG-cap and HA-G3BP1, or the empty vector (EV). Cell lysates were used for immunoprecipitation (IP) with the anti-HA antibody. IP samples were analyzed by western blotting. **(B)** Cap inhibited the interaction of endogenous cGAS and G3BP1. PK15 cells were transfected with FLAG-cap or the empty vector. The cell lysates were used for IP with the anti-cGAS antibody, and the IP products were western blotted. The results represent data from three independent experiments, ***p < 0.001.

Based on the results of our previous experiments, we suspected that Cap may block cGAS recognition of DNA by interacting with G3BP1. Therefore, HEK 293T cells were co-transfected with FLAG-Cap, FLAG-cGAS, HA-G3BP1, or empty vector, and at 24 hpt, the cell lysates were prepared and a co-IP assay was performed using anti-HA followed by immunoblotting. As shown in Figure 5A, both cGAS and Cap co-precipitated with G3BP1 and Cap reduced the amount of cGAS co-precipitated by G3BP1. To verify this, we performed an endogenous experiment where HEK 293T cells were co-transfected with FLAG-Cap or empty vector. The cell lysates from them were prepared at 24 hpt, and a co-IP assay was conducted using anti-cGAS followed by immunoblotting. As shown in Figure 5B, Cap also inhibited the interaction of endogenous cGAS and G3BP1, indicating that the interaction between Cap and G3BP1 reduced the interaction between cGAS and G3BP1, thereby preventing cGAS from recognizing intracellular DNA.

### Overexpression of G3BP1 Reduces the Inhibition Activity of Cap on cGAS-STING Signaling

**Figure 6:**
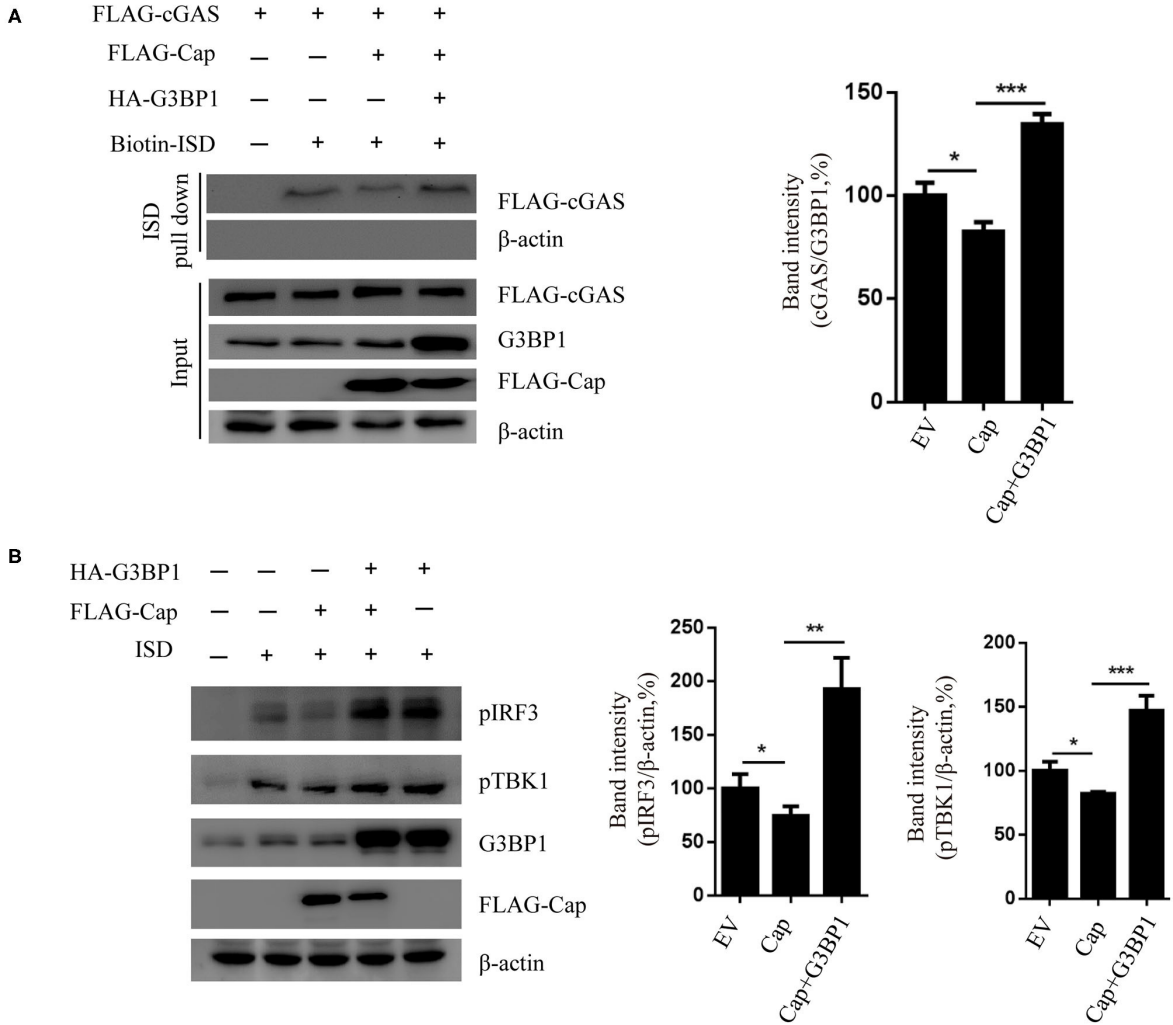
G3BP1 reduces the inhibition activity of Cap on cGAS-STING signaling. **(A)** Cap inhibited ISD binding to cGAS and overexpression of G3BP1 reversed this effect. HEK 293T cells co-transfected with indicted vector and transfected with biotin-ISD (2 μg/well) 24 h after the vector transfection. The cell lysates were used for DNA pull-down assays and western blotting. **(B)** Cap inhibits TBK1 and IRF3 phosphorylation, whereas G3BP1 has the opposite effect. PK15 cells were transfected with the expression vector indicated and then transfected with ISD (2 μg/ml) 24 h after the vector transfection. The cells were lysed and used for western blotting analysis. The results represent data from three independent experiments, *p < 0.05; **p < 0.01; ***p < 0.001.

To investigate whether G3BP1 is involved in Cap inhibition of the cGAS-STING-TBK1-IRF3 pathway, HEK 293T cells were co-transfected with FLAG-cGAS, FLAG-cap, or HA-G3BP1. At 24 hpt, the cells were transfected with biotin-ISD for another 2 h and the cell lysates were prepared from them for biotin-ISD pull-down and immunoblotting analysis. The results showed that Cap inhibited ISD binding to cGAS, and overexpression of G3BP1 reversed this effect (Figure 6A). We next overexpressed G3BP1 and Cap and found that Cap inhibits the TBK1 and IRF3 phosphorylation induced by ISD, but the overexpression of G3BP1 increased the phosphorylation levels of TBK1 and IRF3 (Figure 6B). These results indicate that G3BP1 promotes DNA binding to cGAS and stimulates the TBK1-IRF3 pathway, and overexpression of G3BP1 reduces the inhibition activity of Cap on cGAS-STING signaling.

## Discussion

cGAS is an important cytosolic sensor for DNA, and the cGAS-STING pathway is very important for host defenses against viral infections (24). cGAS recognizes foreign DNA via a dimeric cGAS-DNA complex that synthesizes cGAMP from ATP and GTP, and cGAMP subsequently binds and activates STING. Activated STING then recruits TBK1 to phosphorylate IRF3 and activate IKK to phosphorylate IκBα, and STING then activates the transcription factors NF-κB and IRF3 to induce type I IFN production (16, 17, 26, 27). It has been reported that PCV2 promotes IFN-β production through the cGAS-STING signaling pathway (16). However, many DNA viruses have acquired various effective mechanisms for blocking such host defenses (18, 28–30). For example, protease VP24 of herpes simplex virus 1 inhibits ISD-mediated production of IFN-β by abrogating the interaction between TBK1 and IRF3, thereby inhibiting the activation of IRF3 by blocking its phosphorylation and dimerization (31). ORF52 of Kaposi's sarcoma-associated herpesvirus directly inhibits cGAS enzymatic activity to prevent cGAS-mediated DNA-sensing signals (18, 32). One study showed that dengue virus inhibits type I IFN production in infected cells by cleaving human STING (29). Another reported that PCV2 disrupts the interaction of karyopherin alpha 3 (KPNA3) with p-IRF3 and blocks p-IRF3 translocation to the nucleus, thereby reducing the mRNA level of IFN-β and IFN-β promoter activity driven by the cGAS-STING-IRF3 pathway (33). Additionally, PCV2 ORF5 enhances viral replication by dampening type I IFN expression (34).

PCV3 is a novel circovirus that can cause clinical diseases similar to those caused by infection with PCV2, and because the infections caused by PCV3 are globally quite widespread, gaining better understanding of it is important (35). We previously found PCV3 Cap protein inhibits type I IFN signaling via its interaction with STAT2 (23), but very few studies have been conducted on the host's immune response to PCV3. Whether PCV3 can inhibit the production of interferon in other ways is unclear. Studies on the effects of other DNA viruses on the host immune response can provide a reference. In recent years, the cGAS-STING signaling pathway has become a new research hotspot for host resistance to pathogens. Many DNA viruses can hijack this pathway to evade innate immunity. In the present study, we found that PCV3 Cap inhibited DNA-induced IFN-β mRNA transcription and activation of the IFN promoter. Therefore, we speculated as to whether Cap might affect the production of IFNs through the cGAS-STING pathway, and our further research found that Cap suppressed cGAS binding to DNA. To further investigate the mechanism whereby PCV3 Cap inhibits IFN production, we used IP and mass spectrometry assays to screen for the cellular interacting partners of Cap. G3BP1 was found to interact with Cap, and the RGG domain of G3BP1 is necessary for the interaction with Cap. G3BP1 was confirmed to function by promoting DNA binding and activating cGAS (22), and many viruses have been shown to target G3BP1 and inhibit SG formation (21). We have reached a similar conclusion in the present study. Knockdown of G3BP1 significantly reduced ISD-induced TBK1 phosphorylation, indicating that G3BP1 is involved in ISD-induced IFN promoter activation. Cap was found to inhibit the interaction between cGAS and G3BP1, and overexpression of G3BP1 reduced the inhibition activity of Cap for cGAS-STING signaling. Collectively, these results indicate that Cap can influence the cGAS-STING pathway and inhibit type 1 IFN production.

In conclusion, PCV3 Cap inhibits cGAS recognition of DNA by interacting with G3BP1, thereby affecting the production of IFN-β directed by the cGAS-STING pathway (Supplementary Figure 1).

## Data Availability Statement

The datasets presented in this study can be found in online repositories. The names of the repository/repositories and accession number(s) can be found below: https://www.ncbi.nlm.nih.gov/, KY418606.

## Author Contributions

PZ, HS, ZX, and CS designed the research. The experiments were performed mainly by HS and some experiments were performed with the assistance of PZ, ZX, XL, SW, and YL. HS analyzed the data. PZ wrote the manuscript. All authors contributed to the article and approved the submitted version.

## Conflict of Interest

HS was employed by company Wen's Foodstuff Group Co. Ltd. The remaining authors declare that the research was conducted in the absence of any commercial or financial relationships that could be construed as a potential conflict of interest .

## Supplementary Material

Supplementary Figure 1 Model showing how Cap antagonizes the action of IFNs. Schematic showing the mechanism underlying Cap inhibition of the cGAS-STING signaling pathway. The interaction between Cap and G3BP1 competes with cGAS for binding to G3BP1 and prevents cGAS from recognizing DNA, thereby inhibiting the production of IFN.
