# Supplementary figures and images for: Porcine Circovirus Type 3 Cap Inhibits Type I Interferon Induction Through Interaction With G3BP1
Source: Front Vet Sci. 2020 Dec 17;7:594438. doi: 10.3389/fvets.2020.594438 (PMC7773638; doi:10.3389/fvets.2020.594438)

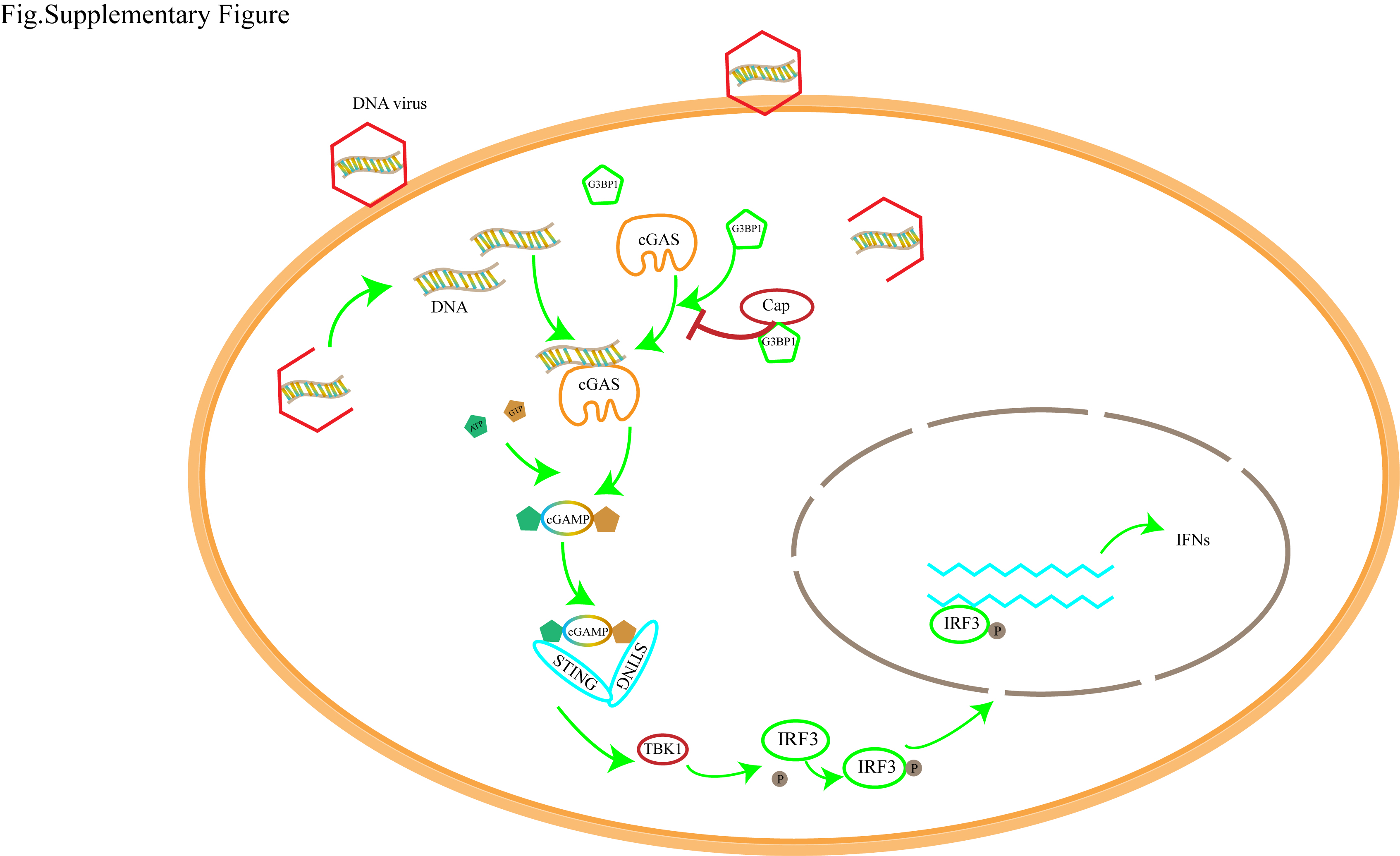

Supplement: Supplementary Figure 1 — Model showing how Cap antagonizes the action of IFNs. Schematic showing the mechanism underlying Cap inhibition of the cGAS-STING signaling pathway. The interaction between Cap and G3BP1 competes with cGAS for binding to G3BP1 and prevents cGAS from recognizing DNA, thereby inhibiting the production of IFN. [file Image_1.JPEG]
